# Surgery of congenital breast asymmetry—which objective parameter influences the subjective satisfaction with long-term results

**DOI:** 10.1007/s00404-021-06218-0

**Published:** 2021-09-03

**Authors:** Vivien Noisser, Andreas Eigenberger, Maximilian Weiherer, Stephan Seitz, Lukas Prantl, Vanessa Brébant

**Affiliations:** 1 University Centre for Plastic, Aesthetic, Hand and Reconstructive Surgery, University Hospital Regensburg, Franz-Josef-Strauß-Allee 11, 93053 Regensburg, Germany; 2 Faculty of Mechanical Engineering, Ostbayerische Technische Hochschule Regensburg (OTH Regensburg), Regensburg, Germany; 3 Regensburg Medical Image Computing (ReMIC), Ostbayerische Technische Hochschule Regensburg (OTH Regensburg), Regensburg, Germany; 4 Department of Obstetrics and Gynecology, Caritas Hospital St. Josef, University of Regensburg, Regensburg, Germany

**Keywords:** Congenital breast asymmetry, Poland syndrome, Lipofilling, Silicone implant, 3D volumetry, Breast Q^™^

## Abstract

**Purpose:**

Congenital breast asymmetry is a serious gynecological malformation for affected patients. The condition hits young women in puberty and is associated with socio-esthetic handicap, depression, and psychosexual problems. Surgical treatment is usually early in the patient's lifetime, so a long-term sustainable solution is important. Although postoperative outcome has been evaluated in several studies before, this study is the first to analyze which objective parameters have the greatest influence on subjective satisfaction with long-term results.

**Methods:**

Thirty-four patients diagnosed with congenital breast asymmetry that underwent either lipofilling or implant therapy between the years of 2008 to 2019 were examined. On average, our collective comprised patients seven years after surgery. Data were mainly gathered through manual measurements, patient-reported outcome measures (Breast Q^™^), and breast volumetry based on 3D scans (Vectra^®^ H2, Canfield Scientific).

**Results:**

Among all analyzed parameters, only areolar diameter correlated significantly negatively with the subjective outcome satisfaction of the patient. Regarding the subjective assessment of postoperative satisfaction with similarity of the breasts, again the mean areolar diameter, but also the difference in areolar diameter and breast volume between the right and left breasts correlated significantly negatively.

**Conclusion:**

Areolar diameter was revealed as being a significant factor influencing subjective long-term satisfaction in breast asymmetry patients. Moreover, 3D volumetry proves to be an effective tool to substantiate subjective patient assessments. Our findings may lead to further improvements to surgical planning and will be expanded in further studies.

## Introduction

The female breast plays an essential role in defining the human silhouette. Not the size of the breast is crucial for esthetics, but the overall appearance [1], which is largely determined by symmetry [2]. Congenital breast asymmetry thus represents a serious malformation to patients with the condition, with young women in puberty particularly affected. In this phase of life, humans are usually vulnerable, self-critical of their bodies, and anxious of how they compare with their peer group [3]. The breast is considered as a symbol of femininity, attractiveness, sexuality, and fertility. Any deformation of the breast is associated with a reduction in or loss of these values and is often viewed as a socio-esthetic handicap. Especially psychological symptoms such as depression and psychosexual problems burden the patients [4–7]. However, it is important to note that breast asymmetry to some degree is natural, and small differences in volume between the right and left breasts may be detected in nearly all women [8–10]. In contrast, obviously recognizable breast asymmetry should be classified as a deformity [8]. The specificity of the patient group lies in the phenotyping during puberty [11, 12]. As a direct result of the described impairments, women with breast asymmetry feel a strong urge to undergo plastic surgery [3]. Most experts recommend early correction to avoid both emotional and social problems, such as stigmatization [11, 13]. In several studies, affected women demonstrated a significant improvement in their subjective psychological stability after surgical correction. In addition, even in undressed situations, an increase in self-esteem has been reported [3, 14]. In general, treatment takes place relatively early on in the patient's life, so a long-term and sustainable solution is of particular importance to the patient [12].

Asymmetrical breasts still present a major challenge in plastic surgery [4]. Since the patient and the surgeon may evaluate a favorable outcome differently [15, 16], this study analyzes which objective factors influence subjective long-term satisfaction in patients with congenital breast asymmetry.

## Materials and methods

### Recruitment and participants

Prior to participant recruitment, the ethics committee of Regensburg University approved the study (approval number: 20-1654-101). Patients diagnosed with breast asymmetry and who underwent correction surgery involving either lipofilling or silicone implant at our institution (University Center for Plastic, Aesthetic, Hand and Reconstructive Surgery, Regensburg) between the years of 2008 and 2019 were included in the study. Women who were minors at the time of the retrospective data collection, had epilepsy, had undergone mastectomy, or patients with acquired breast asymmetry were excluded from the study. The data were collected between March and July 2020. A compact overview of our patient collective is portrayed in Table 1.

**Table 1:** Description of our patient collective

*n* = 34	Mean (± SD)	Range
Age (at time of data collection) [in years]	30 (± 5.8)	21–45
Age (at time of first breast surgery) [in years]	21 (± 5.6)	16–42
BMI (at time of last breast surgery) [in kg/m^2^]	23.6 (± 4.1)	18–38
Length of postoperative period [in years]	7 (± 3.3)	0.9–12
Cup size	C	A–E
Scar quality [given a scale from 1 (best) to 3 (worst)]	1.4 (± 0.6)	1–3
Number of surgery sessions	2 (± 1.2)	1–5

Half of the patients were treated with lipofilling and the other half with silicone implant augmentation. Our cohort included five women diagnosed with Poland syndrome, fifteen patients with tuberous breast deformity and breast asymmetry, thirteen women with Amazon’s syndrome, and one patient with chest deformity. At the time of surgery, ten out of 34 patients were minors. At the time of data collection (on average seven years later), all 34 patients were adults.

### Study design

Prior to participating, all patients provided their informed consent.

**Fig. 1:**
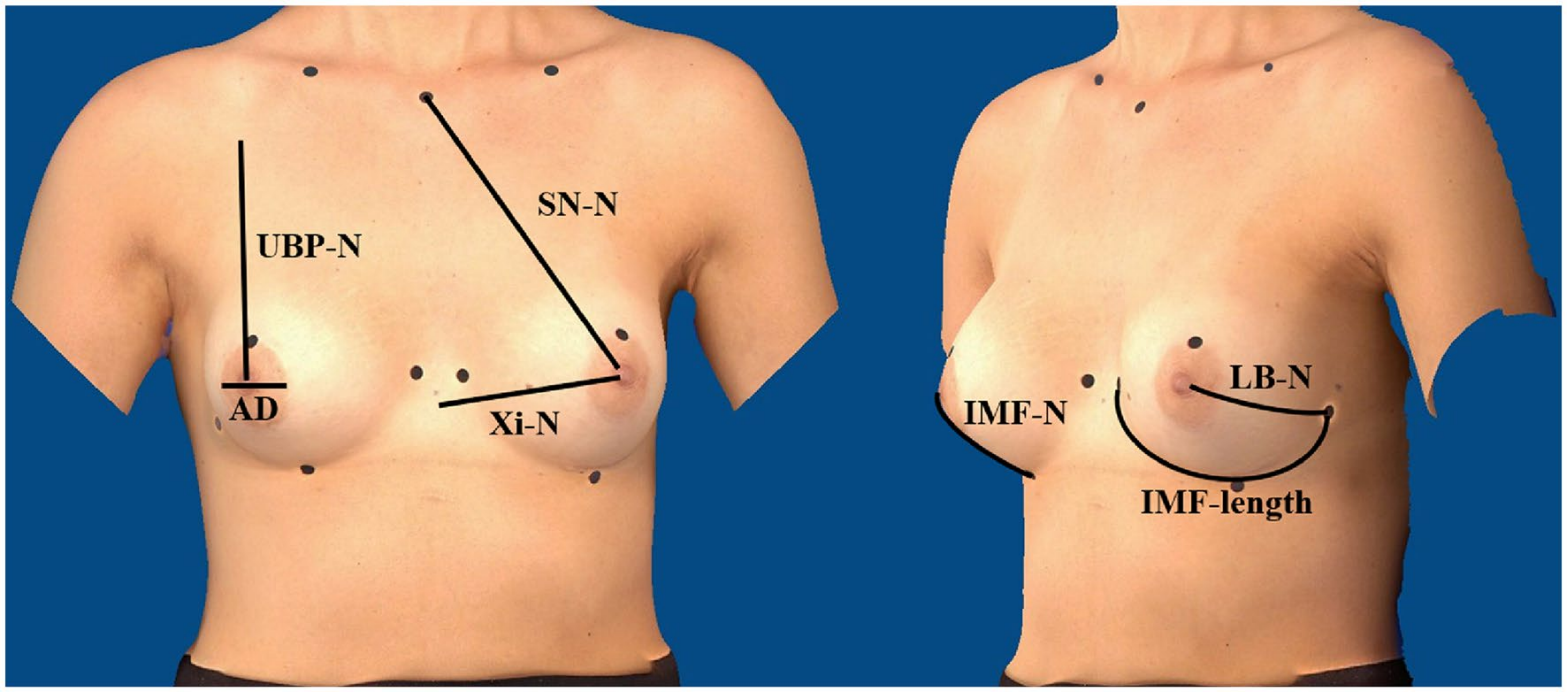
Detailed illustration of measurements (picture created with Canfield Vectra^®^ H2 and edited), patient with implant therapy

Data for the following objective and subjective parameters were collected during clinical examination. The cup size (A/B/C/D/E) was determined by medical assessment. The scar quality was evaluated on a scale from 1 to 3 (hardly/moderate/highly visible scars). As body measurements can be an important predictor of female attractiveness [17], the patient was measured manually with a classic tape measure along the skin surface. The following measurements were recorded: sternal notch to nipple (SN–N), inframammary fold to nipple (IMF–N), upper breast pole to nipple (UBP–N), xiphoid to nipple (Xi–N), lateral breast pole to nipple (LB–N), inframammary fold length (IMF-length) and areolar diameter (AD). Figure 1 depicts the recorded measurements, which were taken as the shortest distance along the skin surface.

### Three-dimensional volumetry

**Fig. 2:**
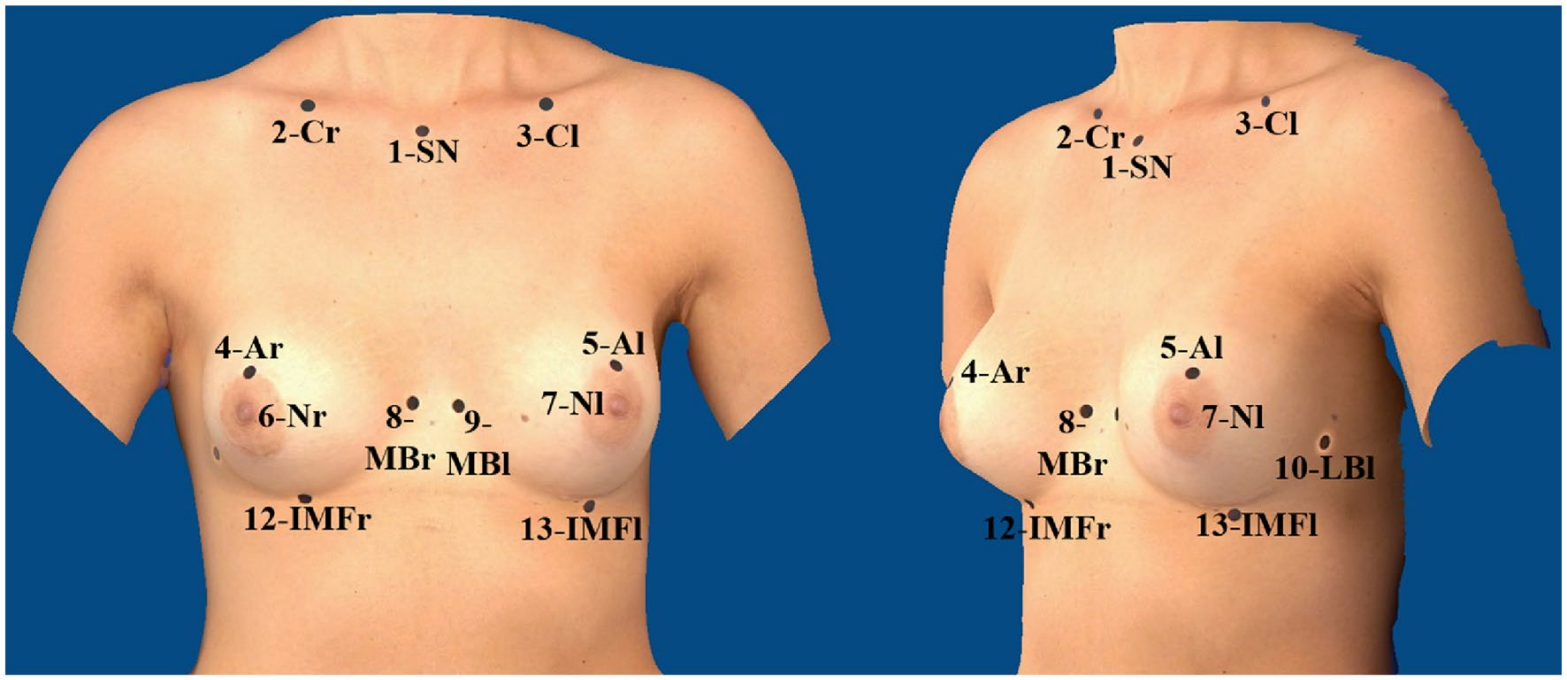
Reference points for Canfield Vectra^®^ H2 (picture created with Canfield Vectra^®^ H2 and edited), patient with implant therapy

Three-dimensional breast volumetry was performed with the portable Vectra^®^ H2 (Canfield Scientific, USA), which is frequently used in the literature [18–26]. The 3D model can be analyzed in terms of breast volume and various breast dimensions by using the Breast sculptor^®^ software package. Based on a modified protocol, O'Connell et al. [20] conducted a validation study using the Vectra^®^ XT, in which the anterior axillary line was selected as the lateral breast boundary, see Fig. 2. We integrated these findings, in order to achieve the best possible reproducibility in our study:

(1) Sternal notch (SN), (2) center of the right clavicle (Cr), (3) center of the left clavicle (Cl), (4) most cranial point of the right areola (Ar), (5) most cranial point of the left areola (Al), (6) right nipple (Nr), (7) left nipple (Nl), (8) end of right medial inframammary fold (MBr), (9) end of left medial inframammary fold (MBl), (10) end of right lateral inframammary fold (LBr), (11) end of left lateral inframammary fold (LBl), (12) most caudal point of the right inframammary fold (IMFr), (13) most caudal point of the left inframammary fold (IMFl).

Points (8) and (9) are seen as the medial border of the breast. They are more precisely defined as the point of the inframammary fold with the shortest distance to the anterior median line. The lateral border of the breast is marked by points (10) and (11). As the appearance of the breast varies greatly from woman to woman [27], depending on the shape of the breast, the inframammary fold can be highly variable and might end diffusely [28], which makes reproduction of the present reference point inaccurate. The high inaccuracy of reference points was also criticized by O'Connell et al. [20]. They defined the lateral breast boundary as the anterior axillary line [20]. To use a reproducible reference point and to achieve uniformity with manual volumetry, we decided to follow O'Connell et al. [20] and defined the point of the lateral breast boundary as the intersection of the anterior axillary line with a line through the nipple.

All images were taken at our institute, processed and overlayed, and analyzed using Vectra^®^ and Breast Sculptur^®^. The camera equipment includes a special positioning mat, precisely specifying the position of the patient and photographer for each of the three images. The 45° angle of the arms was checked with a goniometer. In addition, a telescopic stick was used to assist the patient with maintaining the requested position of the arms fixed. The first and third images capture the patient at a 45° angle, from the right and left, respectively. The second image is taken frontally. The Vectra^®^ software then assembles a 3D model from the images. In order to perform calculations on the resulting 3D model, 13 reference points are required. As the automatic detection of the reference points by the Vectra^®^ software did not work as well as expected, the reference points were marked on the patient before the images were taken and then set accordingly in the software. This seems to be a common problem, which is well described by other researchers [19]. Following Eder et al. [29], we used the positive effects of pre-marking the reference points [29].

### Patient-related-outcome measures: Breast Q^™^ questionnaire

All participants completed the Breast Q^™^ questionnaire (Breast Q Version 2.0^©^, Augmentation Modules Pre- and Postoperative Scales, German (DE) Version, The University of British Columbia, licensed for non-profit users by Memorial Sloan Kettering Cancer Center and translated by Mapi Research Trust, 2008). The Breast Q^™^ is a standardized, patient-reported outcome measure (PROM) on subjective quality of life, developed by Pusic et. al. [30]. Over five years, Breast Q^™^ was validated with the help of about 3000 women [31]. It is thus considered a clinically relevant, standardized outcome evaluation instrument that meets psychometric criteria and captures the patient's self-assessment according to a state-of-the-art system [31]. Recently, Breast Q^™^ was also used by other researchers in similar studies [32, 33].

This study implemented the module probing the patient’s satisfaction after breast surgery. It evaluates the satisfaction with the breast surgery outcome by means of eight questions with three possible answers for each: 1 = disagree; 2 = somewhat agree; 3 = completely agree. Using the enclosed transformation score, the sum for each module may be interpreted directly as a value between 0 (worst value) and 100 (best value), effectively providing a percentage “score of satisfaction”.

## Statistical analysis

Statistical evaluation was carried out with SPSS^®^ Statistics Version 25.0.0. from IBM^®^. Using Spearman's correlation, all meaningful parameters were examined for any correlation with patient subjective outcome satisfaction. Spearman’s correlation was chosen because either at least one parameter was scaled ordinally, or at least one outlier was found. A significance level of 0.05 was considered.

## Results

### Correlations of objective parameters with long-term subjective satisfaction

The results of the Breast-Q^™^ questionnaire are portrayed in Table 2.

**Table 2:** Spearman’s correlation of outcome satisfaction with surveyed parameters

*n* = 34	Breast Q^™^ satisfaction with outcome	
	r_s_	*p* value
Age (at time of data collection) [in years]	0.260	0.137
Age (at time of first breast surgery) [in years]	0.000	0.999
BMI (at time of last breast surgery) [in kg/m^2^]	− 0.179	0.310
Length of postoperative period [in years]	− 0.80	0.653
Cup size	− 0.136	0.443
Scar quality (on a scale from 1 (best) to 3 (worst))	− 0.113	0.523
Number of surgery sessions	0.234	0.183

r_s_ = correlation coefficient

*Significant (*p* < 0.05)

**Highly significant (*p* < 0.01)

As seen, none of the objective parameters investigated correlates significantly with long-term outcome satisfaction.

The results for the manually measured anthropometric distances are depicted in Table 3.

**Table 3:** Results of Spearman’s correlation for body measurement parameters (mean and ∆ between the right and left breasts), objective volumetry, and symmetry index with outcome satisfaction or satisfaction with similarity of breasts

*n* = 34	Breast Q^™^ satisfaction with similarity of breasts		Breast Q^™^ satisfaction with outcome	
	r_s_	*p *value	r_s_	*p *value
MV SN–N	− 0.159	0.370	− 0.300	0.084
MV IMF–N	0.071	0.689	− 0.119	0.504
MV UBP–N	− 0.194	0.272	− 0.039	0.826
MV Xi-N	− 0.115	0.519	− 0.186	0.293
MV LB–N	− 0.305	0.079	− 0.153	0.387
MV AD	− 0.355*	0.039	− 0.405*	0.017
MV IMF-length	− 0.168	0.343	− 0.314	0.071
$\Delta$ SN–N	− 0.269	0.123	0.098	0.580
$\Delta$ IMF–N	− 0.137	0.441	− 0.043	0.811
$\Delta$ UBP–N	− 0.187	0.290	− 0.054	0.761
$\Delta$ Xi-N	− 0.287	0.100	− 0.283	0.105
$\Delta$ LB–N	0.002	0.989	− 0.176	0.320
$\Delta$ AD	− 0.381*	0.026	− 0.242	0.168
$\Delta$ IMF-length	0.076	0.669	− 0.138	0.435
$\Delta$ 3D volume	− 0.389*	0.023	− 0.231	0.189
Satisfaction with similarity of breasts	1	0	0.597**	< 0.01

r_s_ = correlation coefficient

*Significant (*p* < 0.05)

**Highly significant (*p* < 0.01)

Specifically, we tested whether a distance (mean value (MV) or difference (∆) between the right and left sides) had any influence on subjective outcome satisfaction or the perception of breast similarity in the long-term observation. The latter is a single question concerning the patient's subjective satisfaction with the similarity of her breasts (on a scale from 1 (worst) to 4 (best)).

Regarding the mean values of the areolar diameter (MV AD), there was a significant correlation between both the general satisfaction with the result (r_s_ = − 0.405; *p *value = 0.017) as well as the subjective assessment of similarity between the right and left breasts (r_s_ = − 0.355; *p* value = 0.039). No other mean values of the body measurements (SN–N, IMF–N, UBP–N, Xi–N, LB–N, IMF-Length) had any significant influence on the subjective satisfaction result or the self-perception of similarity between both breasts. The larger the mean value of the areolar diameter, the more dissatisfied the patients were with the long-term result and the more dissimilar they felt their two breasts to be.

Regarding the difference between the right and left breasts (∆), we only found significant correlation for the ∆ in the areolar diameter (∆ AD). This correlated negatively with the patient's subjective satisfaction with the similarity of her breasts (*p *value = 0.026; r_s_ = − 0.381). Clearly, the smaller the difference between the areolar diameters of the right and left breast, the more satisfied the patient was with the similarity of her breasts. In addition, the question considering satisfaction with similarity of the breasts correlated strongly with the overall satisfaction with outcome. The *p* value was less than 0.01 and the correlation coefficient 0.597. We conclude that the satisfaction with the similarity of the breasts has a huge impact on the satisfaction with the overall, long-term result. None of the other calculated values for ∆ of the body measurements (SN–N, IMF–N, UBP–N, Xi–N, LB–N, IMF-length) had any significant influence on the resulting satisfaction or the satisfaction with the similarity of the breasts.

Finally, the smaller the volume difference between the right and left breasts, the more satisfied the patient was with the similarity of her breasts (*p* value = 0.023; r_s_ = −0.389).

## Discussion

### Discussion of methods

In comparison with other authors in this field who tend to assess patient satisfaction between a few months and a maximum of one year postoperatively [14, 24, 34], we examined the patients in our study on average seven years postoperatively. This made it possible to attach comparatively greater importance to the long-term outcome. However, the disadvantage of such long-term observation lies in generation of the study collective. Although our study collective of 34 patients is not unusual compared to similar studies of congenital breast asymmetry (Kuzbari et al. [35] *n* = 30; Neto et al. [14] *n* = 35; Eder et al. [29] *n* = 28), the small collective should be viewed critically, as it allows only limited generalizations. Therefore, a multicenter approach may prove advantageous in follow-up work in order to generate a larger collective.

We assessed long-term outcome satisfaction using the validated Breast Q^™^ questionnaire, which was developed specifically for breast augmentation [30] and corresponds to the current state of research [36]. Especially in earlier studies, some authors worked with a simple satisfaction scale [10, 35, 37, 38] or used measurement tools that did not specifically refer to the breast [14]. In recent years, however, the breast-specific Breast Q^™^ has increasingly replaced these and is used frequently in research [24, 32–34]. Critically, however, it should be noted that long-term outcome satisfaction as assessed by the Breast Q^™^ is a very complex construct [39] and may be influenced by many other individual factors besides those assessed in this study. For instance, many people consider plastic surgery as a cure for their personal and relationship problems [40]. This may have created a bias in long-term outcome satisfaction. Another drawback of the Breast Q^™^ is that the sensitivity of the breast after surgery is not included in the questionnaire. However, this appeared to be particularly relevant in our study. Some patients reported numbness or hypersensitivity as one of their biggest postoperative problems. We, therefore, recommend including breast sensitivity in follow-up work.

By employing a state-of-the-art 3D scanning technique to assess objective outcome, we base the methodology of our study on the latest research. In the current literature, state-of-the-art 3D volumetry has been evaluated as both objective and effective [41, 42]. Regarding volumetry with Vectra^®^ H2 from Canfield, however, it should also be noted that there are some limitations. The camera is well suited to creating a 3D model of small to medium-sized breasts and to analyzing their volumes. However, for larger and ptotic breasts, the most caudal point of the inframammary fold, an essential reference point for Canfield's Vectra^®^ H2 (see methods), is not visible and obscured by the breast itself. This limited proper volumetry in this study, as was the case for Koban et al. in 2018 [41]. In summary, the methodology of our study is characterized not only by its proximity to the current state of research, but also by the comprehensive survey of subjective and objective factors and the long-term observation of the rare diagnosis of congenital breast asymmetry.

### Discussion of results

**Fig. 3:**
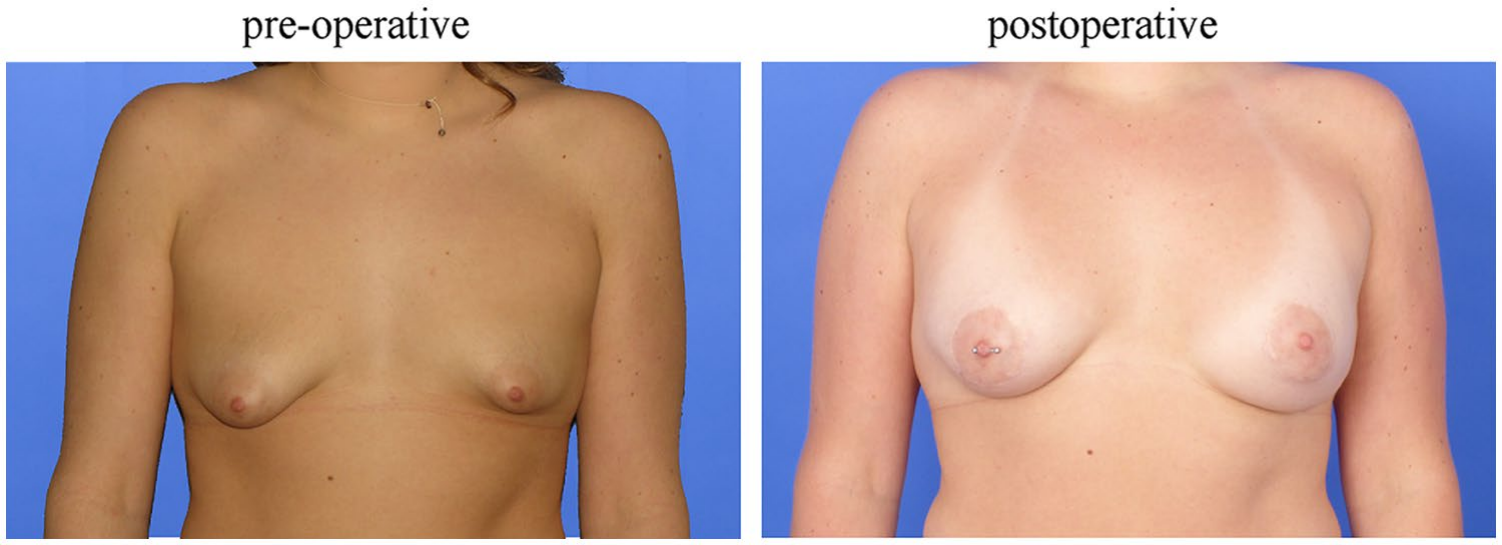
Patient with congenital breast asymmetry (tubular deformity): the left image was taken prior to surgery in 2017. The right image was taken in 2020, therapy: implant

**Fig. 4:**
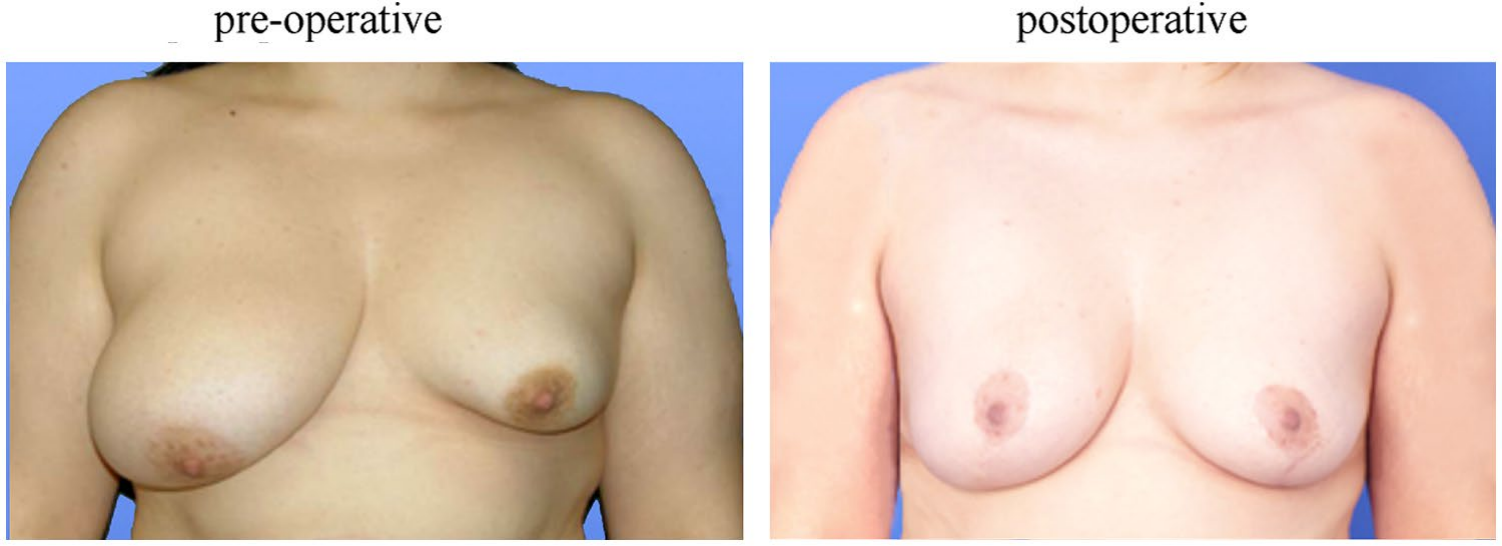
Patient with breast asymmetry (Poland syndrome): the left image was taken prior to lipofilling therapy in two sessions (surgery: 2010 and 2013). The right image was taken in 2020

In our study, volumetry with the Vectra^®^ H2 by Canfield proved to be an effective tool to quantify a patient’s subjective satisfaction in a side-by-side comparison of the right and left breasts (Figs. 3 and 4). We were thus able to confirm the results of Ji et al. [42] proving the 3D-scan technique to be an objective and effective tool for analyzing and documenting breast morphology (Figs. 3 and4). In addition, we were also able to support the results of Eder et al. [29] regarding the increasing relevance of 3D technology in the comparison of breast augmentation therapies with this study.

We found the mean value of the areolar diameter to be remarkable, as it correlated significantly with the patient's subjective assessment of similarity between their breasts as well as with the long-term outcome. Furthermore, our study confirmed the findings of Osinga et al. [38] that neither scar appearance nor breast size had any significant influence on the overall outcome. In everyday clinical practice, the SN–N distance is considered the most important parameter. However, we did not reveal that a smaller ∆SN–N had any positive effect on the subjective symmetry score for the patient. Our results differ from Osinga et al. on this aspect [38]. The mean areolar diameter (MW AD) was the only body measurement in our work that had a significant influence on the long-term outcome. With this finding, our study supports the results of Pietruski et al. [43], who recently demonstrated in their eye-tracking study that the nipple-areola complex is one of two key focus areas of the female breast.

## Conclusion

The strength of our study lies in its employment of a state-of-the-art 3D-scanning technique with its accuracy of data collection and reliable registration compared to other methods. The combination of subjective and objective criteria in the evaluation of the outcome underlines the comprehensive approach of our study. The long-term follow-up design of our study is unique in this specific field of congenital breast deformity. It is a real advantage considering the young age of the patients at the time of surgery. Areolar diameter was revealed as being a significant influencing factor in patient-subjective long-term satisfaction for breast asymmetry patients, which will be a focus of our future research and could lead to further improvement in surgical planning. One limitation of our study is the rather small collective, which is justified by the rare clinical picture and the long-term retrospective focus of the study. We thus plan to focus on multicentric studies, in order to generate a larger collective. Furthermore, a prospective design could help us to answer further questions considering breast sensitivity.

Our study takes us one step closer to the long-term goal of establishing robust instruments to evaluate the results of breast surgery and contribute to the quality assurance of breast surgery in congenital breast asymmetry.
